# Poly(vinylidene fluoride) and Carbon Derivative Structures from Eco-Friendly MOF-5 for Supercapacitor Electrode Preparation with Improved Electrochemical Performance

**DOI:** 10.3390/nano8110890

**Published:** 2018-11-01

**Authors:** Krzysztof Cendrowski, Wojciech Kukulka, Tomasz Kedzierski, Shuai Zhang, Ewa Mijowska

**Affiliations:** Nanomaterials Physicochemistry Department, Faculty of Chemical Technology and Engineering, West Pomeranian University of Technology, Szczecin, Piastów Ave. 42, 71-065 Szczecin, Poland; wojciech_kukulka@zut.edu.pl (W.K.); tomek_k95@o2.pl (T.K.); Shuai.Zhang@zut.edu.pl (S.Z.)

**Keywords:** MOF-5 synthesis, MOF-5 synthesis from recycled DMF, supercapacitors, MOF-5 carbonization, electrode preparation

## Abstract

Electrodes from carbonized Zn_4_O(1,4-benzodicarboxylic acid) (MOF-5) structures were prepared successfully via evaporating the solvent with a poly(vinylidene fluoride) (PVDF) binder. The solvent used for a nanocomposite cast was easily removed. Such an elegant method for preparing electrodes provides a facile, cost-effective, and void/cracking-free nanocomposite distribution on the current collector. The highly porous nanoparticles containing pure carbon attach well to the PVDF membrane which results in an increased active surface area of the electrode to 847 m^2^/g. The electrochemical analysis shows that the best weight ratio of CMOF-5 to PVDF equals 85:15, 80:20, and 75:25, respectively. The specific capacitance of these samples is 218 F/g, 210 F/g, and 180 F/g, correspondingly. An additional advantage of the electrode prepared from the carbonized MOF-5 is the possibility to synthesis MOF structures from recovered substrates used in its synthesis (distilled N,N-Dimethylformamide DMF and terephthalic acid recovered from polyethylene terephthalate waste). We will demonstrate this in this contribution as well. Furthermore, the carbonized MOF-5 can be recovered from the spent electrode and reused again in the electrochemical device.

## 1. Introduction

A novel type of energy storage material based on carbon with high power density and long cyclic life has attracted attention due to its wide applications [1,2]. Activated carbons were the most studied electrode material for supercapacitors due to their large specific surface area, tunable pore size, commercial availability, and acceptable cost [3,4,5]. The perfect material should have both a high capacitance and a good rate capability to possess high energy and power density. Carbon materials with high capacitance should have a large specific surface area in which micropores (<2 nm) are predominant. Recently, the synthesis of porous carbon with a controlled size of micropores and mesopores has been reported. It was obtained by the carbonization of metal-organic frameworks (MOFs). A detailed investigation of MOF-5 thermal decomposition revealed that carboxylic bridges between benzene rings and Zn_4_O clusters break down and zinc oxide crystals, CO_2_, benzene, and amorphous carbon are produced [6]. From all of the carbonized zinc-based MOFs, MOF-5 shows the highest porosity and surface area with potential applications as a supercapacitor electrode material [7]. The control over the carbonization parameters is crucial for the successful transformation of the metal-organic framework into porous carbon. According to Liu et al., the surface area of the carbonized MOF-5 (CMOF-5) depends on the thermal conditions of the MOF-5 carbonization and ranges from 1521 m^2^/g to 2542 m^2^/g [8]. Additionally, during the MOF-5 thermolysis, the spherical and rod-like ZnO nanoparticles are produced, which cause a deterioration of the surface area of the CMOF-5 (carbon structure cracking and voids formation) [9]. Recent studies showed that zinc oxide nanoparticles exhibit potential application as a material for electrodes preparation in supercapacitors [10] and sensors for detecting CO concentration [11].

The disadvantage in applying the MOF-5 at a higher scale is the high amount of the N,N-Dimethylformamide (DMF) waste used in synthesis. DMF is categorized as toxic to the natural environment and can cause serious health problems and birth defects in humans [12,13]. Therefore, the amount of waste containing DMF (produced and released to the environment) should be reduced to the minimum. For that reason, the DMF was recycled after synthesis of MOF-5 in order to show the possibility of reducing the amount of toxic waste if applied in the industry.

The reuse of other waste, like polymer bottles, will increase the possibility to transfer the MOF-5 synthesis to a higher scale and potential MOF-5 industrial application. Synthesis of the MOF structure from the converted waste plastic was previously studied and proposed. The polyethylene terephthalate (PET) structure is composed of terephthalic acid, that can be released under hydrothermal conditions in an autoclave. The synthesis of MOFs structure from recovered terephthalic acid was reported by Yu-Ting Huang et al. who reported the successful synthesis of NTHU-2, NTHU-3, MOF-5, MIL-53 and MIL-101 from the recovered acid linker [14]. Willem P.R. Deleu et al. reported the synthesis of MIL-53 (based on aluminum) and MIL-47 (based on vanadium) using recovered terephthalic acid [15]. A similar approach to the synthesis of MOF structures from recovered acid linker was reported by Jianwei Ren et al. who described PET hydrolysis and Cr-MOF synthesis simultaneously [16].

Poly(vinylidene fluoride) (PVDF) is an attractive material in electrochemistry applications. The advantages of this material include its high polarity, a high dielectric constant, a low degree of crystallinity, morphology control through binary and ternary polymer/solvent phase diagram, good wettability and electrochemical stability due to the presence of a strong electron-withdrawing group (C–F). It can be used as a polymer electrolyte, a separator membrane, or a binder material for preparing of carbon electrodes [17,18,19]. The last of the listed applications is the most common. Due to the low plasticity of carbon materials, an additional binder is required. The durability of the carbon-based electrodes depends on the material plasticity that varies with the structure and size. The PVDF electrodes are usually made by dispersing studied material in the polymer solution and mixing continuously until it has evaporated. Then, the obtained powder is pressed into tablets and dried under a vacuum [20]. Different weight ratios of PVDF are used as a binder to the active material. According to the literature, the content of the binder in the electrode is in the range of 5 wt% to 20 wt% [21,22,23]. The composition of binder and the conductive and active material has a significant effect on the electrochemical results. For many years, researchers have been looking for the most effective binder material and the final composition of the tested electrodes [24,25].

In this study, polymer-CMOF-5 electrodes were fabricated using the PVDF polymer. The effects of polymer binder content on the electrochemical properties were investigated in comparison to the electrodes prepared by a pressing pellet. In order to characterize the electrochemical properties of electrodes, cyclic voltammetry (CV), galvanostatic charge-discharge (GCD), and electrical impedance spectroscopy (EIS) measurements were measured. The novelty of the conducted investigation consists of three issues: (i) MOF-5 synthesis from recycled terephthalic acid and DMF, (ii) the reuse of active material obtained from used electrodes, and (iii) systematic studies on correlation between electrode composition (amount of binder material) and electrode morphology structure and its electrochemical performance. There are reports on the synthesis of MOF structures from recycled terephthalic acid, but we have proven that the correct MOF-5 structure can be also obtained with recycled DMF. This allows for the development of a new PET waste management strategy and limits the use of DMF, which is very harmful to humans and the environment. The reuse of active material from spent electrodes will also reduce the substrate consumption and make aware that electrodes do not have to be disposable. It turns out that the electrode obtained from recycled active material still exhibits very good electrochemical properties. Finally, we also performed a very detailed investigation over the optimal composition of electrodes. We have defined a very precise range of active-binding material ratios to obtain the best electrochemical properties. We also showed what is happening with the morphology of electrodes and their electrochemical properties at extreme ratios. This will allow us to prepare electrodes in the future with an optimal structure and the best electrochemical results.

## 2. Materials and Methods

### 2.1. Materials

Hydrochloric acid (36%) and N,N-Dimethylformamide (DMF) were purchased from Chempur (Piekary Śląskie, Poland). Zinc nitrate hexahydrate (Zn(NO_3_)_2_·6H_2_O) and Terephthalic acid (C_6_H_4_(COOH)_2_) were provided by Sigma Aldrich (MERCK, Darmstadt, Germany).

### 2.2. Preparation of Carbonized MOF-5

MOF-5 was prepared according to the previous reports [9]. Briefly, 1.65 mmol of zinc nitrate hexahydrate and 0.89 mmol of terephthalic acid were dissolved in 2.34 mol N,N-Dimethylformamide (DMF). The mixture was sonicated for 1 h. The obtained solution was transferred into the autoclave and stirred for 48 h at a temperature of 150 °C. Then, the obtained suspension of MOF-5 was removed from the autoclave and put aside to precipitate the solid particles and then the excess of solvent was removed.

The MOF-5 suspension was transferred to a ceramic boat and placed in the center of a tube furnace. Next, the furnace was heated from room temperature up to 1000 °C. This temperature was maintained for 1 h with an Ar flow of 100 mL/min. Further, it was purified with concentrated hydrochloric acid.

### 2.3. Electrochemical Characterization

The electrodes were prepared by the dispersion of the CMOF-5 and poly(vinylidene fluoride) (PVDF) in acetone. The obtained suspension was spread all over the surface of the current collector and allowed to evaporate at room temperature. The electrodes with comparable mass were separated by glassy fibrous paper (Whatman GF/G) soaked with electrolyte.

The electrodes used for comparison, with a diameter of 9 mm and a mass of 2 mg, were pressed from a mixture of CMOF-5 (90%), PVDF (5%) and acetylene black (5%). Electrodes with a comparable mass were separated by glassy fibrous paper (Whatman GF/G) and placed between the current collectors.

The capacitance of materials was determined using the two-electrode Swagelok® type cell in the voltage range from 0 V to 1.0 V with 1M H_2_SO_4_ as the electrolyte. The voltammetry experiments were conducted at a scanning rate from 1 mV/s to 200 mV/s and a galvanostatic charge/discharge at a current density from 1 A/g to 60 A/g was used to estimate the specific capacitance C in farads (F) per gram of active materials on one electrode. The voltammetry results were presented as a function of C = f (E) and VMP3 multichannel generators (Bio-logic, Seyssinet-Pariset, France) were used for taking measurements.

### 2.4. Determining Specific Capacitance

In order to evaluate the charge storage capacity quantitatively, the specific capacitance of active material was calculated according to the following equation:

C = 2IΔt/(mΔV)(1)

In this equation, I stands for current, Δt is discharge time, m is the mass of the active material in a single electrode, and ΔV is the voltage change during discharge. This equation is the most widely used expression to calculate the specific capacitance and to determine whether a material is suitable to be used in an electrode [26].

### 2.5. Characterization Techniques

A thermogravimetric analysis (TGA; TA Instrument, New Castle, DE, USA) was conducted under argon flow with a heating rate of 10 °C/min using a TA Instrument SDT Q600. To investigate the crystal composition of the samples, X-ray diffraction (XRD; X’Pert PRO Philips diffractometer, Co. Ka radiation, Almelo, Holland) patterns were carried out using an X’Pert Philips Diffractometer with a Cu anode (Kα1 = 1.54056 Å). The structure and morphology of the samples was analysed with a transmission electron microscope (TEM; Tecnai F30, Thermo Fisher Scientific, Waltham, MA, USA) and scanning electron microscopy (SEM; VEGA3 TESCAN, Brno, Czech Republic) equipped with a spectroscopic analysis modulus—X-ray energy dispersive (Bruker, Billerica, MA, USA). The N_2_ adsorption/desorption isotherms were acquired using a Quadrosorb SI (Quantachrome Instruments, Boynton Beach, FL, USA). The specific surface area was calculated by the Brunauer-Emmett-Teller (BET) method. An atomic force microscopy Nanoscope V MultiMode 8 (Bruker, Billerica, MA, USA) was employed to examine the mechanical properties of membranes.

## 3. Results and Discussion

**Figure 1:**
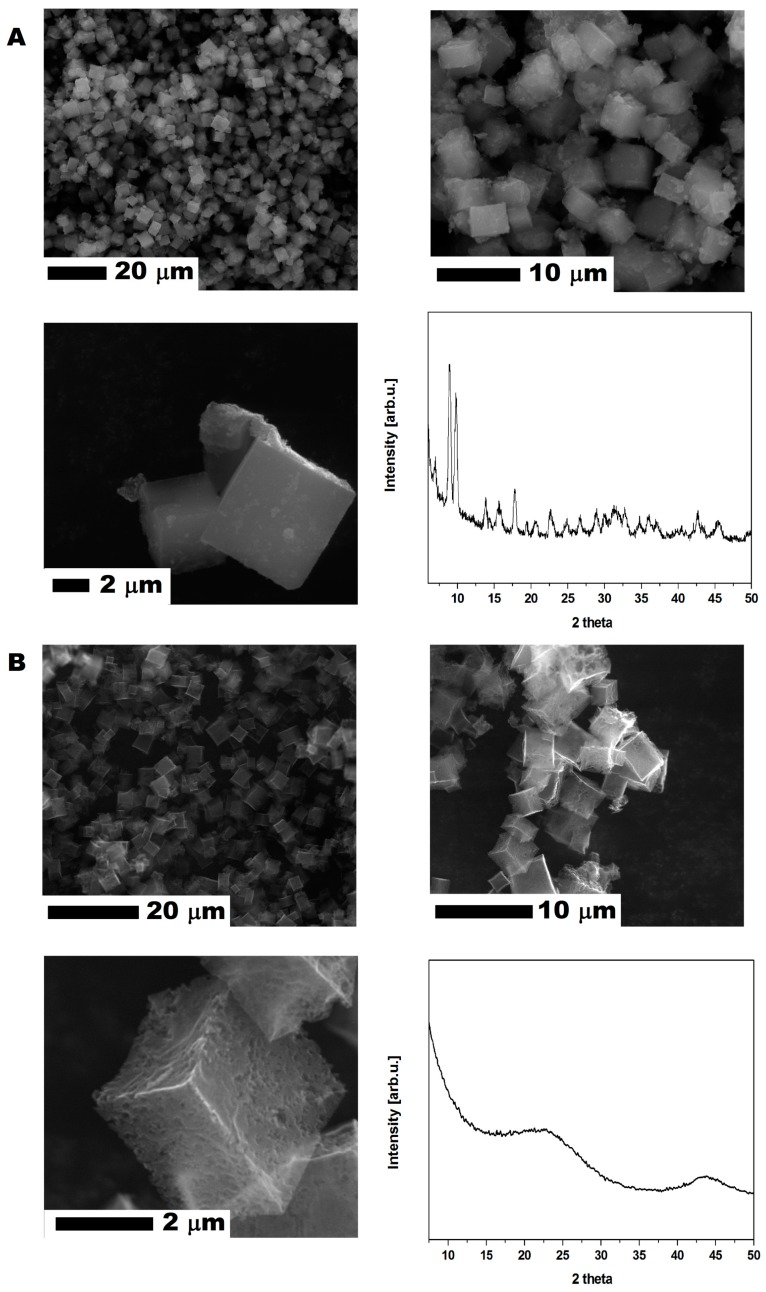
The SEM images of MOF-5 before (**A**) and after (**B**) carbonization with X-ray diffraction patterns.

CMOF-5 was produced according to the previously described method [9]. The MOF-5 (Figure 1A) crystal and CMOF-5 (Figure 1B) structures present cube-shaped particles. The MOF-5 crystals have a dense morphology in contrast to CMOF-5, which is transparent. The scanning electron microscopy (SEM) images also revealed that the surface of the obtained crystals is smooth and without any pores. After carbonization, a porous structure with additional cracks and cavities (from the removed zinc oxide) was observed [9].

The crystal structure of the prepared MOF-5 and CMOF-5 was confirmed by X-ray diffraction (XRD). As presented in Figure 1, the XRD pattern of pristine MOF-5 shows four characteristic reflections at 7.00° (002), 9.78° (022), 13.81° (004), and 15.62° (024) with a 2θ angle [27] according to the standard card (MOF-5 ref. Code. SAHYIK, CCDC-256965).

The MOF-5 carbonization (at a high temperature of 1000 °C) results in decomposing MOF-5 to organic ligands and zinc oxide. Organic ligand carbonization increases the reduction of ZnO content by carbon and Zn evaporation. Having evaporated Zn, CMOF-5 showed a broad peak between 20° and 25°, as well as at ~45°, which corresponds to the disordered nature of graphite.

**Figure 2:**
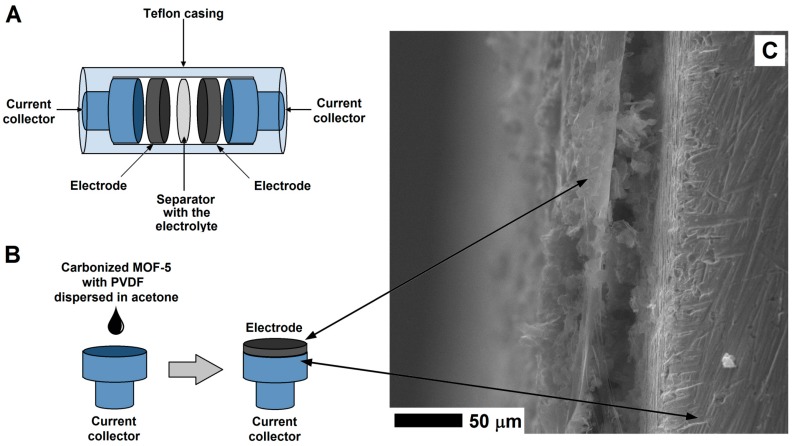
The structure of supercapacitor (**A**) and electrode preparation (**B**) with the SEM image of the electrode on the surface of the current collector (**C**).

The experimental process of preparing electrode from the CMOF-5 and PVDF is presented in Figure 2. In order to prepare an electrode, CMOF-5 was dispersed in acetone by ultrasound sonication. The resulting suspension was dropped onto a current collector and evaporated at room temperature. When the solvent was evaporating at room temperature, the current collector was covered with the PVDF/CMOF-5.

**Figure 3:**
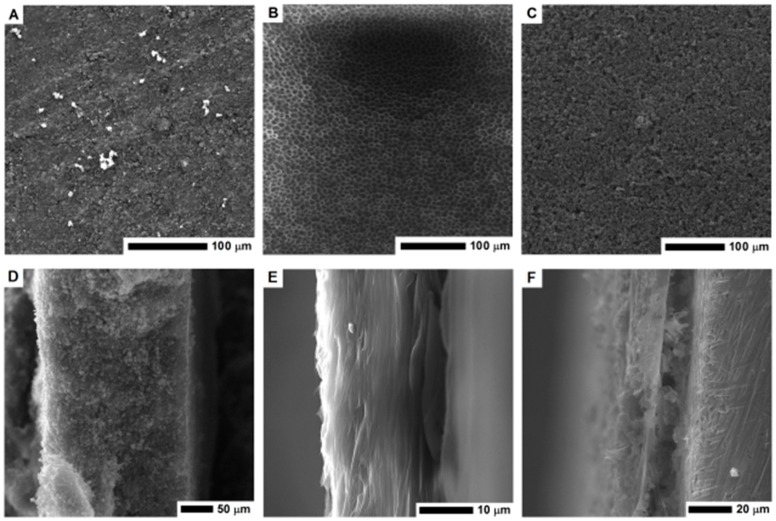
The SEM images of the surface area and cross-section of the MOF-5 pellet (**A**,**D**); PVDF layer (**B**,**E**) and nanocomposite of PVDF and carbonized MOF-5 (**C**,**F**).

Scanning electron microscopy was employed to examine the electrode morphology—cross-section and surface structure (Figure 3). The structure of PVDF membrane is heterogeneous, it consists of a thin network of thicker pores in a form of open-celled foams. The PVDF membrane exhibits a typical asymmetric structure with micropores. As clearly seen in Figure 3E, the cross-section of the PVDF membrane reveals the structure with ultra-thin layers. The thickness of the membrane is about 10 μm.

The surface of the electrode formed by pellet pressing and its cross-section is shown in Figure 3A,D respectively. Its cross section is free of any micropores, which limits the electrolyte penetration and interaction deeper in the structure. When the CMOF-5 and PVDF nanocomposite was formed, the pellet became more compact and thicker. Higher magnification images of the electrode surface are presented in the Supplementary materials (Figure S1). The pellet thickness was estimated based on the SEM image (~230 μm).

The PVDF/CMOF-5 electrode, cross-section (Figure 3F), and surface structure (Figure 3C) was visualized using SEM. The thickness of the electrodes attached to the current collector was about 30–50 μm. The electrolyte interaction layer was connected with the current collector through PVDF-CMOF bridges. Without adding CMOF-5, the surface of the PVDF membrane is flat. Once CMOF-5 was added, nanomaterials formed columns and stacks as well as covers and penetrated the polymer, hence, a rough surface is obtained (Figure 3C). Compared to the pellet morphology, the surface area of the PVDF and CMOF-5 membrane should be larger due to its rough and porous surface and hollow structure present under the surface layer. Thus, more ions from the electrolyte may be received, as a result, the capacitance should be higher.

The positive aspect in electrode prepared by the evaporation is not only their thinner thickness but increased cohesion and lack of electrostatic interactions. Electrostatic charge and cohesion of the electrodes are crucial when supercapacitors are assembled. In supercapacitors made from pressed pellets, electrostatic charge can be accumulated on the supercapacitor casing—Swagelok PFA tube fitting. It can cause carbon material breakage and sticking to the supercapacitor tube fitting. Additionally, thinner electrodes tend to break during supercapacitor assembly. These problems are eliminated when the electrodes are formed by evaporation on the surface of the current collector.

The surface area of the sample prepared by high pressure compressing PVDF/CMOF-5 nanocomposite reach 706 m^2^/g with a pore volume of 1.3 cm^3^/g and an average pore radius of 7.3 nm. In cases where the electrode is formed during evaporation, a higher surface area is obtained (847) m^2^/g. The increased surface area of the nanocomposite is related to its higher pore volume: 1.5 cm^3^/g. The average pore radius of the electrode formed during evaporation is similar to the sample formed by the compression (7.3 nm). The nitrogen adsorption isotherm of electrodes with CMOF-5 are presented in the Supplementary information (Figure S2). The nitrogen adsorption isotherms were observed to be type IV, similarly to CMOF-5 [22].

**Figure 4:**
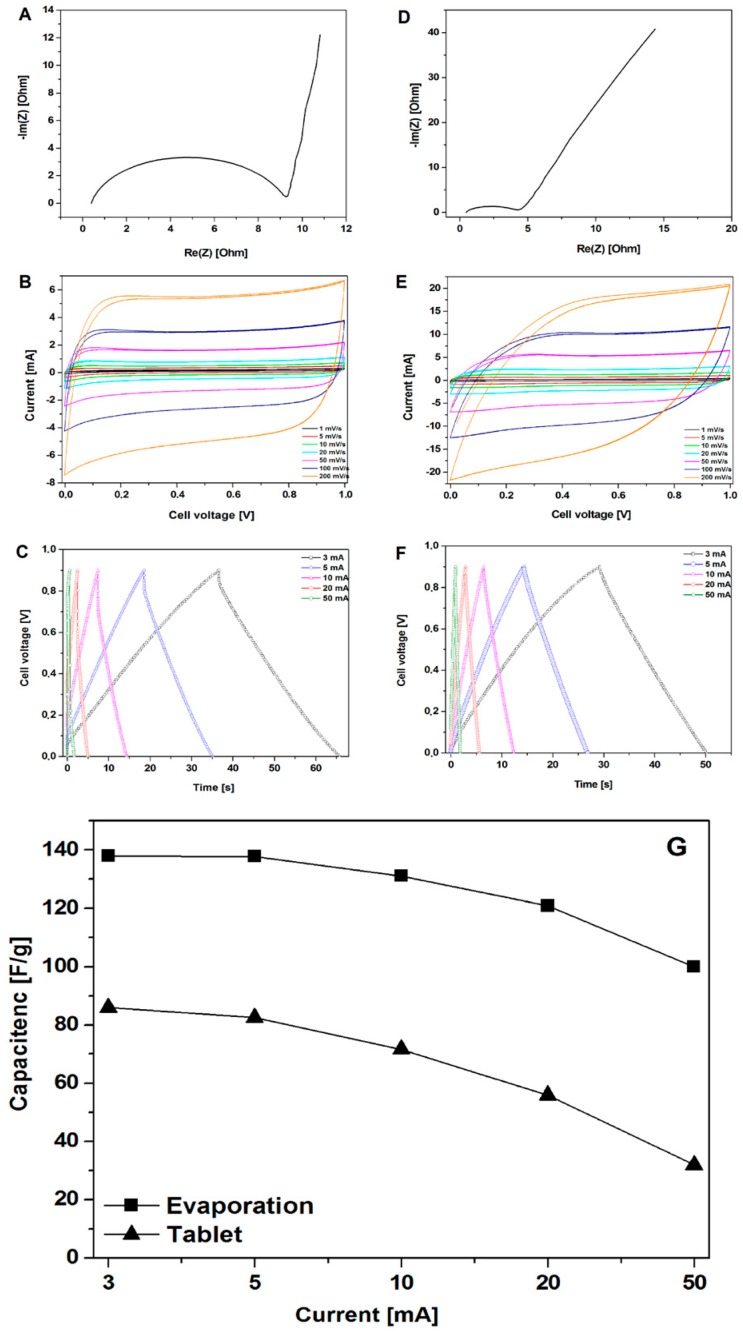
The Nyquist plots, CV, and GCD curves of the electrodes prepared by evaporation (**A**–**C**) and pellet formation (**D**–**F**) along with the measured specific capacitance (calculated from the GCD curves) for the electrodes prepared by evaporation and pellet formation (**G**).

The electrochemical performances of the CMOF-5 materials were investigated by cyclic voltammetry, galvanostatic charge-discharge, and electrochemical impedance spectroscopy (EIS) via a two-electrode system in a 1 M H_2_SO_4_ electrolyte (Figure 4). The capacitive properties of all the carbon electrodes were measured in the potential range of 0–1 V. The electrochemical properties of the electrodes, obtained in a manner of evaporation and forming pellets, were compared.

Figure 4A,D show the EIS results of CMOF-5, where the frequency dependent impedance is given as the real (Z′) and imaginary (Z″) components in the Nyquist plot. At low frequencies, the vertical curve was featured and it showed nearly ideal capacitive behavior [28]. Obviously, for the CMOF-5 electrode prepared via the evaporation method, a more vertical nature appears at lower frequencies.

Cyclic voltammetry is a suitable tool for estimating the difference between the Faradaic and non-Faradaic reactions. Figure 4B shows the CV curves of CMOF-5 electrodes prepared by evaporation in the 1M H_2_SO_4_ aqueous electrolyte at varied scan rates from 1 mV/s to 200 mV/s. The rectangular shapes without any redox peaks indicate the excellent conductivity with a high capacitive current as EDLCs. However, the CV curve shows distortion at a high scan rate of 200 mV/s, which can be attributed to the limited mass transfer or ionic transport at high rates. As a control, the CV curves of the same electrode material prepared in the form of pellets (Figure 4E) shows a smaller integral area, which indicated a much lower capacitance. As a result, it proved that the evaporation method is more efficient in the enhancement of specific capacitance.

Figure 4C shows typical galvanostatic charge-discharge curves of the CMOF-5 electrodes prepared by evaporation at various current densities from 3 A/g to 50 A/g. The regular triangular shapes suggest that the electrodes possess similar ideal capacitive performance and better electrochemical reversibility. CMOF-5 electrodes show good coulombic efficiency and an ideal behavior for electrochemical supercapacitor. Moreover, the linear dependence of the discharge potential vs time was determined. It implied no major Faradaic process [29]. The calculated specific capacitance of CMOF-5 was 138 F/g at a current density of 3 A/g, while the specific capacitance was still relatively high (99.99 F/g) at a higher current density of 50 A/g with the retention of 72.5 %. The capacitance of the CMOF-5 electrode prepared by the pressing method was also calculated in order to compare it to the evaporation method (Figure 4F). The capacitance of 86 F/g at 3 A/g was much lower than the value in the evaporation method.

Figure 4G showed the comparison of capacitance at different current densities between the evaporation and pressing method. Due to the technical limitations in the preparation of the electrodes, it was impossible to obtain a similar thickness of the electrodes. Given the difference in electrodes thickness, it is not possible to sort out the effects of different electrode parameters. However, considering the data on pore size distribution, the results indicate that the evaporation method would form a more suitable space for electrolyte storage, which could increase the capacitance performance. The high specific capacitance and excellent rate capability of CMOF-5 are mainly attributed to their high specific surface area, optimized pore structure, and improved pore volume. An appropriate electrode preparation method could facilitate the diffusion and migration of the electrolyte ion.

**Figure 5:**
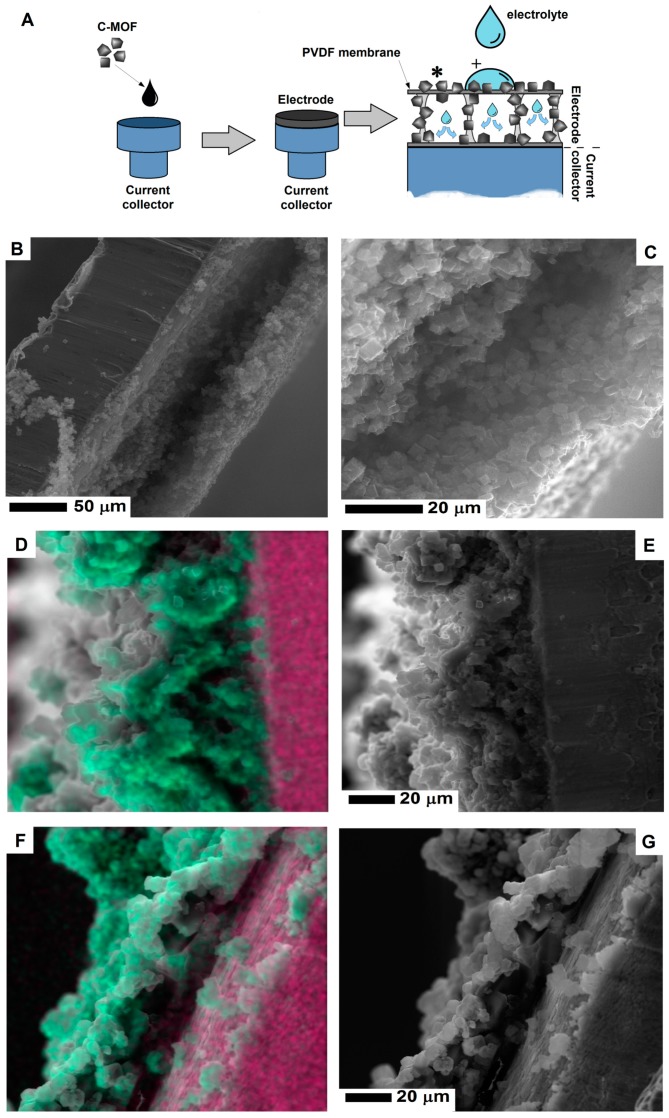
The scheme (**A**) of the electrolyte penetration of electrode and the SEM images of the electrode cross-section before (**B**,**C**) and after electrolyte penetration (**D**,**E** and **F**,**G**). The image in Figure 5D,F show the SEM images with a merge signal from potassium (green) and copper (pink) (To interpret the colors in this figure, the reader is referred to the web version of this article). The experiment scheme shows the spots of elemental mapping presented in Figure 5D,F, marked with (*) and (+) respectively.

To explore how electrolytes penetrated the evaporated electrode, energy dispersive X-ray spectroscopy was applied to verify wetting behavior. The affinity between the electrode and electrolyte is crucial for supercapacitors because electric capacitance is strongly dependent on the surface contact area between the electrode and electrolyte. The model of the penetrating electrolyte through the evaporated electrode is shown in Figure 5A. In order to study the electrolyte penetration, 5M potassium hydroxide was used.

After electrode penetration, potassium hydroxide evaporates and leaves potassium traces. Was determined by EDS mapping. The cross-section of the evaporated electrode before electrolyte penetration is presented in the SEM images (Figure 5B,C). Figure 5E shows the cross-section of the electrode after electrolyte penetration in the inner space of the electrode. After the electrolyte evaporates, the hollow space of electrode is less noticeable due to deposition of potassium hydroxide. Figure 5D shows the merge SEM image of the electrode and the mapping of potassium (marked with green color). Additionally, for a higher contrast between the electrode and current collector, the signal from copper (current collector) was marked with the pink color. In order to prevent any contaminations by potassium contamination (during preparing the electrode cross-section) X-ray signal was collected away from the wetting point of the electrode. Due to electrolyte migration in the electrode, the signal from potassium is detected inside the electrode. Figure 5F,G show the electrode cross-sections collected from the electrolyte wetting point. In contrast to the potassium signal mapping away from the wetting point (Figure 5D), here, the potassium signal is also located on the surface of the electrode. Individual maps of the potassium and copper signal are presented in the Supplementary information (Figure S3).

**Figure 6:**
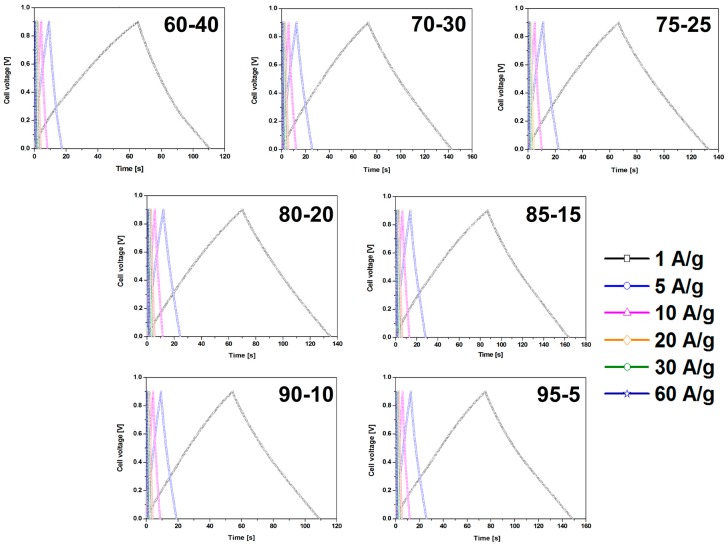
The GCD curves of the electrodes prepared by evaporation with different ratios of CMOF-5:PVDF.

CMOF-5 electrodes prepared by the evaporation method reveal higher capacitance values and smaller retention indicating a better stability than electrodes obtained by pressing. The dependence of the weight ratio of CMOF-5 and PVDF on capacitance results was also investigated. Different weight ratios (95:5, 90:10, 85:15, 80:20, 75:25, 70:30, and 60:40) were checked. All galvanostatic charge–discharge curves of the CMOF-5 electrodes show regular triangular shapes (Figure 6).

**Figure 7:**
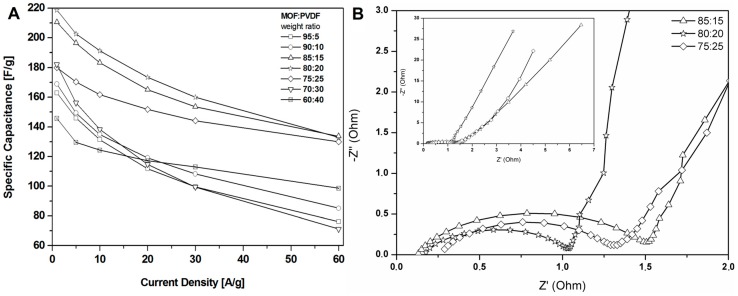
The specific capacitance curves (**A**) and Nyquist plots (**B**) of the electrodes prepared by evaporation with different ratios MOF:PVDF.

The results revealed that a higher content of active material (CMOF-5) provides higher capacitance. However, a higher content of binder material (PVDF) is responsible for the better stability of the electrodes at higher current densities. It is, therefore, necessary to find the optimum weight ratio of the active material and binder to gain the best capacitance and excellent capacitance retention. The most promising samples are observed with a CMOF-5 to PVDF weight ratio equal 85:15, 80:20, and 75:25, respectively. The calculated specific capacitance of CMOF-5-85 and CMOF-5-80 at a current density of 1 A/g is 210.5 F/g and 218 F/g, respectively (Figure 7A). A lower amount of CMOF-5 in respect to the binder results in a decrease of capacitance to 147 F/g for ratio 60:40 at a current density of 1 A/g. The trend of the specific capacitance retention is presented in Figure 7A. The resistance was tested by electrochemical impedance spectroscopy (EIS). Nyquist plots for the three best above-mentioned samples are presented in Figure 7B. The results are consistent with the values of capacitance—the smallest resistance is observed in three samples with a weight ratio of CMOF-5 to PVDF equal 85:15, 80:20, and 75:25, respectively. Their resistance is very low and it is below 1.5 Ω, which indicates a superior conductivity. Nyquist plots of all the samples are presented in Supporting information (Figure S4). The Nyquist plots of CMOF-5-based supercapacitor show an inclined line in the low-frequency region and a semicircle in the high-frequency region. It is known that an ideal capacitor shows a vertical line parallel to the imaginary y-axis [30]. The vertical line in the plots of the samples with a weight ratio between 85:15 to 75:25 at a low-frequency indicates a nearly ideal supercapacitor response.

**Figure 8:**
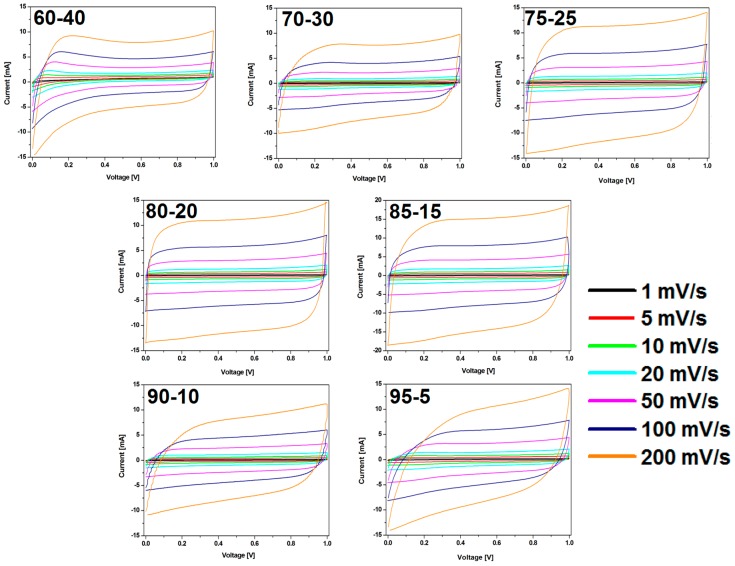
The CV curves of the electrodes prepared by evaporation with different ratios of CMOF-5:PVDF.

The CV results were in good agreement with the observation in charge-discharge curves (Figure 8). The shape of CV curves changes with the weight ratio of CMOF-5:PVDF in the electrode. CMOF-5-80 presents the most rectangular shape without any redox peaks. The sample with the highest content of active material shows a CV slope with a less rectangular shape and looks like a leaf, which indicates a limited mass transfer or ionic transport at high rates and the departure from ideal EDLC behavior.

**Figure 9:**
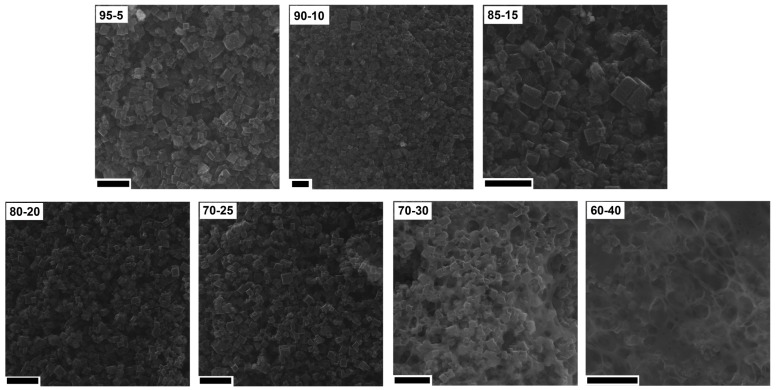
The SEM images of the electrodes prepared by evaporation with different ratios of CMOF-5:PVDF (scale bar 20 nm).

In order to understand the changes in the CV hysteresis loop and specific capacitance, the prepared electrodes were analyzed using SEM (Figure 9). When the content of PVDF was low (from 5% to 20%), cubic shape structures were observed on the surface of the CMOF-5 electrodes. With the further increase in the binder content (25%), the binder excess in the form of layers covering CMOF-5 was rarely visible. The effect of blocking CMOF-5 with the binder is intensified with a higher PVDF content (30%). With a binder content of 40%, the structure of electrodes resembled a membrane structure similar to pure PVDF with CMOF-5 cubic nanostructures.

**Figure 10:**
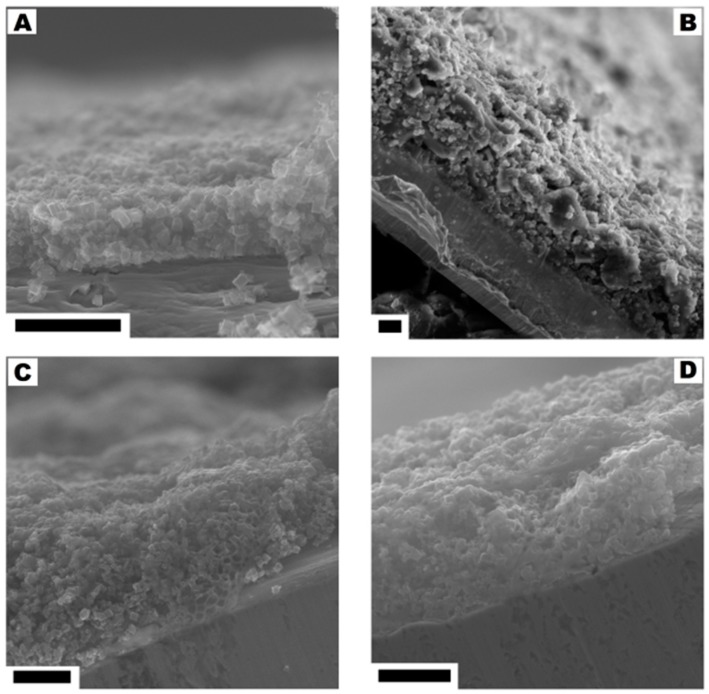
The cross-section of the electrodes corresponding to the CMOF-5:PVDF ratios: from 95:5 to 80:20 (**A**), 75:25 (**B**), 70:30 (**C**) and 60:40 (**D**). The scale bar corresponds to 20 µm.

No significant changes were observed in the cross-section of the electrodes with the ratio from 95:5 to 75:25. With the higher content of the polymer binder (30% and 40%) the structures typical for the polymer membrane started to appear (they were formed during the evaporation of dissolved PVDF). The pore structures that allowed for the direct contact of the electrolyte with the current collector were observed in the SEM images. The comparison of SEM images is presented in Figure 10.

**Figure 11:**
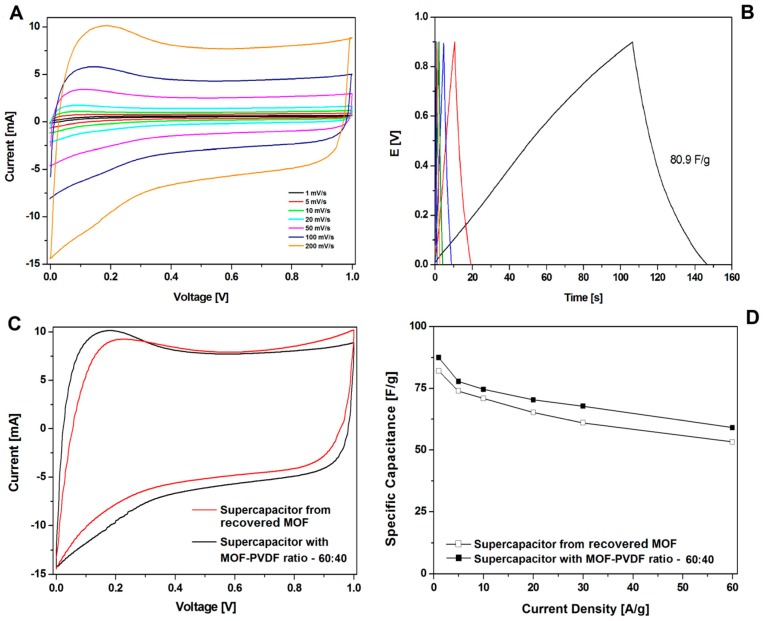
The CV (**A**) and GCD (**B**) curves, correlation of the CV curves at 200 mV/s (**C**) and the GCD capacitance (**D**) of supercapacitors from recycled and pristine carbonized MOF-5 (ratio 60:40).

A great increase in recovering and recycling valuable metals (such as Ni, Co, and Li) [31,32,33,34] and carbon nanomaterials (graphene) [35] from spent electrodes from supercapacitors and batteries is observed. In this contribution, the electrode was prepared with recovered CMOF-5 from previously used electrodes in the weight ratio active material:binder equal 80:20 wt%. The CV hysteresis loop and galvanostatic charge–discharge curves curves are presented in the Figure 11A,B, respectively. The capacitance was lower than the electrode with pristine CMOF-5 (Figure 11D). Additionally, compression of the CV hysteresis loop obtained at a scan rate of 200 mV/s shows characteristic deformations (Figure 11C).

**Figure 12:**
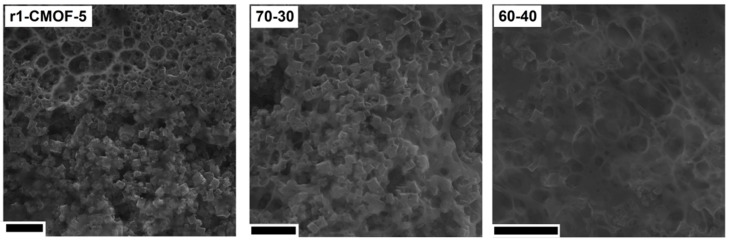
The SEM images of the electrodes from the recycled CMOF-5 and electrodes prepared with ratio 70:30 and 60:40 (scale bar 10 nm).

Further microscopic analysis of the electrode prepared from the r1-CMOF-5 showed that the electrode structure is more similar to the electrode prepared in the ratio of the active material:binder 60:40 than 70:30 (Figure 12). SEM images show the excess of the PVDF binder in the form of a membrane and as a layer covering the CMOF-5 cubic structure.

**Figure 13:**
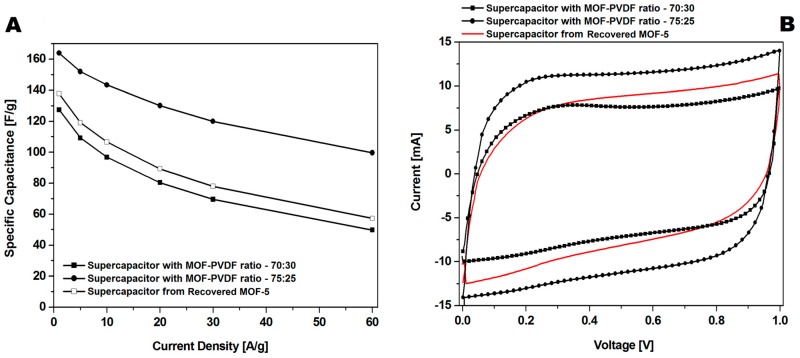
The comparison of the GCD (**A**) and CV (**B**) curves at a 200 mV/s scan rate of supercapacitors made of recovered MOF (additionally purified MOF: r2-CMOF-5) and pristine carbonized MOF (at a ratio of 75:25 and 70:30).

CMOF-5 synthesis requires using expensive toxic solvents. It may lead to a high cost of the nanomaterials production, however, it could significantly reduce the cost by distilling and recycling DMF. Therefore, the potential of cutting the cost through recovery and CMOF-5 recycling from the spent electrodes is an important factor. Therefore, in order to meet this challenge, the optimization of the recovered CMOF-5 purification was continued. The number of acetone purification cycles of the spent electrode was increased from three to five and preceded with washing with concentrated hydrochloric acid. After additional acetone washing and chloride acid purification (r2-CMOF-5), CV hysteresis at a scan rate of 200 mV/s corresponded to the performance of the electrode with a MOF-5 to PVDF ratio between 70:30 wt% and 75:25 wt% (Figure 13B). The r2-CMOF-5 specific capacitance of r2-CMOF-5 increased significantly with respect to r1-CMOF-5 (59 F/g) (Figure 13A).

**Figure 14:**
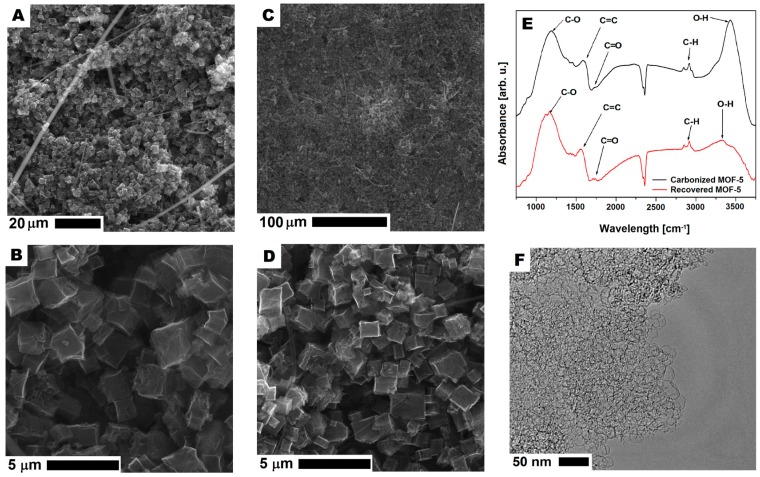
The SEM (**A**–**D**) and TEM (**F**) images of carbonized MOF-5 after recycling and purification. The FT-IR spectrum of carbonized MOF-5 before electrode preparation and after CMOF-5 recovery (**E**).

In order to understand the reason for decreasing the specific capacitance of r2cmo5 with respect to cmof5 (80:20 wt%), the additional analysis of any contamination or structure degradation was performed. SEM images of r2-CMOF-5 showed that in the recovered samples, the cellulose fibers from the separator used in supercapacitors (Figure 14A–D) were present. Moreover, based on the SEM images, no contamination from the polymer binder was detected. This observation was also confirmed by the analysis of the TEM images that showed an open porous structure of CMOF-5 (Figure 14F). The structural stability of recovered and purified CMOF-5 was also confirmed by the FT-IR spectrum analysis that did not show any significant differences (Figure 14E). This analysis showed that the material recovered from the spent electrode could be potentially reused after intense washing from the remaining binder (PVDF) and the additional purification from the cellulose fibers.

The essential aspect of applying the nanomaterial in the industry is not only concerning the control of the structure’s design, stability, and physicochemical properties, but also concerning the economic and environmental concerns. Previous studies on the MOF-5 carbonization provided detailed information on the carbonization mechanism, structure, and the chemical and physical properties depending on the carbonization parameters [9]. Here, there is a focus on the increase in the synthesis yield and reducing the amount of produced waste—DMF. In order to successfully synthetize MOF-5 from the reused DMF, the organic solvent was purified from the unreacted substrates and by-products such as zinc oxide nanoparticles, by distillation. As shown on the SEM images (Supporting information, Figure S5), the structures synthesized from the recovered DMF have a typical MOF-5 cubic structure. The advantage of applying MOF-5 is the possibility to reuse the DMF after the previous process.

**Figure 15:**
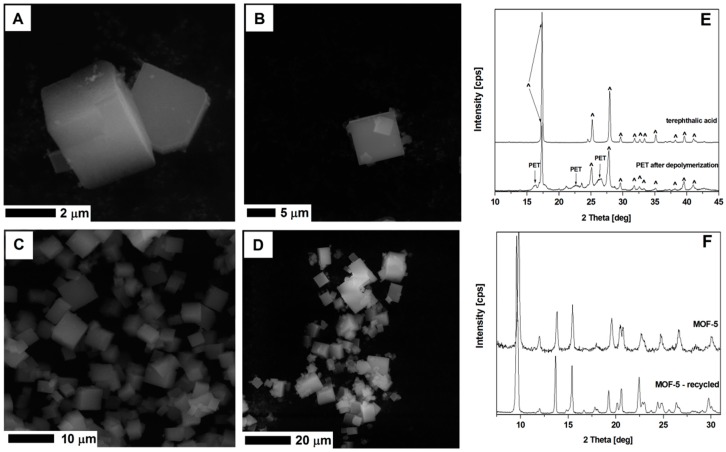
The SEM images of MOF-5 synthesized from fresh substrates (**A**,**B**), recycled DMF and terephthalic acid (**C**,**D**). The XRD spectrum of the terephthalic acid and depolymerized PET (**E**), and MOF-5 from unreacted and recycled substrates (**F**).

The second essential aspect of MOF-5 synthesis is the possibility to reduce the polymer waste like polyethylene terephthalate (PET). The obtained terephthalic acid from depolymerized PET waste (bottles) was successfully used for synthesizing MOF-5. Similar to the previously published studies [13,14,15,16], we were able to recover terephthalic acid by hydrothermal depolymerization PET waste (Figure 15). The MOF structures synthesized using the recovered terephthalic acid shows a typical MOF-5 cubic structure (Supporting information, Figure S1).

MOF-5 synthesized from the recycled DMF and terephthalic acid recovered from the PET waste, show cubic structure, typical for the MOF-5 (Figure 15). The XRD analysis of this sample shows identical peak positions as the MOF-5 structures produced from the virgin substrates. Successful recycling of DMF and terephthalic acid from the PET showed a great potential in the MOF-5 application and transfer of synthesis technology to the industrial scale.

The enhanced electrochemical properties of electrodes with a less dense structure and higher porosity has been already observed in the study of W. Pfleging and J. Pröll [36]. They showed that the laser-generated capillary structures in the electrode materials increased the electrode wetting. Electrochemical cell tests that were performed using unstructured and laser-structured lithium nickel manganese cobalt oxide and lithium manganese oxide demonstrated that the lithium-ion cells containing a laser-structured cathode showed an increased capacity and cell lifetime during cycling [36]. Byeong-Hee Park and Jae-Hwan Choi stated that it is very important to increase the wetted surface area of a carbon electrode for high capacitive deionization performance. An interesting contribution of Park and Choi was the presentation of the electrochemical properties of the electrode from the PVDF-bonded activated carbon powder (ACP). The cyclic voltammetric analysis allowed them to estimate that the specific capacitance reached 74.4–80.3 F/g [37] for the PVDF-ACP electrode, which was similar to our results on the CMOF-5 prepared by pellet compression. The ACP had a comparable surface area, but the electrode (prepared by evaporation) cross-section was far different. The electrode from the PVDF-ACP was covered and bound with a polymer binder. However, the hollow space similar to the PVDF/CMOF-5 electrode was present. The method of electrode preparation proposed by Meng-Fang Lin and Pooi See Lee includes PVDF as a polymer binder in the form of a membrane with barium titanate. The in situ synthesis approach proposed by Lin and Lee enabled the formation of oxide nanoparticles in the presence of the grafted polymer with a hydroxyl functionalization group for direct coupling with oxide nanofillers [38].

Bin Xu et al. proposed hierarchical porous carbons with a high surface area, easily prepared from poly(vinylidene fluoride) (PVDF) by NaOH activation and one step carbonization. NaOH introduced to the synthesis system acted as an interceptor of HF obtained from the PVDF decomposition, as well as an activation agent. Microscopic and nitrogen adsorption measurements (SEM, TEM, and BET) revealed that carbon has a highly developed hierarchical porous structure containing a wide range of interconnected pores (micropores, mesopores, and macropores) and a high surface area. The electrochemical performances of the PVDF-based porous carbon in 1 mol/L Et4NBF4/AN non-aqueous electrolyte showed a similar specific capacitance to our membrane—141 F/g (for the NaOH:PVDF ratio 3:1). The Bin Xu et al. method used PVDF for preparing carbon, but obtained an electrode that still required polymer binder [39].

Kenji Takahashi et al. showed the mechanical degradation of graphite/PVDF composite electrodes. According to the reported data, a micrometric thick PVDF film showed a similar mean Young’s modulus despite the carbon nanomaterial addition (2.0 Gpa and 1.9 Gpa) [40]. The average Young’s modulus value for the PVDF film with and without C-MOF-5 addition is 57 GPa and 51 Gpa. However, the samples with a conductive material addition (graphene) showed a lower Young’s modulus value of 1.9 GPa/2.0 GPa due to the thinner layers (13–17 μm). The Young’s modulus values of the binder samples (PVDF with conductive material addition) submerged in the electrolyte solution were found to be approximately five times smaller than in the case of the dry samples. The same trend was observed in tensile strengths values of wet samples with respect to the dry samples. Therefore, dry samples should start with high Young’s modulus. They reported that when using dry samples, the Young’s modulus values were different from the wet Young’s modulus values. Kenji Takahashi et al. reported data proving the higher potential of the electrodes prepared by evaporation over the pressed one. Electrochemical tests showed that the evaporation electrode preparation outperformed the pellet compressed electrodes and that it was due to the different inner structure of electrodes. Except for the enhanced specific capacitance, the evaporated electrodes showed a high Young’s modulus, higher durability, and easier method for manufacturing.

The current state of the art is listed in Table 1. Our data show a similar capacitance to the literature data.

## 4. Conclusions

In summary, the results proved that CMOF-5 may be a material for forming electrodes using the suspension of PVDF/acetone in the coating process. The presented data show that the technique used for electrode preparation is very important and responsible for their specific capacitance. The electrode prepared by evaporation possessed a higher capacitance than the electrode prepared by pressing due to the enhanced electrolyte penetration and electrode interaction. The enhanced specific capacitance was caused by the formation of a thin electrode with accessible and connected highly porous, carbonized MOF structures. Except for the method for preparing electrodes, the binder and active material ratio that allow for the successful CMOF-5 binding with an enhanced specific capacitance are highly important. The presented data showed that an excess of the active material did not increase the electrode specific capacitance. The optimum content of the active material (85:75 wt%) increased the specific capacitance allowing it to bind and transfer charge between the nanostructures. When below the optimum content of the active material, the excess of the binder clogged the pores in the CMOF-5 structure, thus, decreasing the electrode specific capacitance.

CMOF-5 materials present a straightforward approach to produce porous carbon electrodes and open new avenues for other applications. Except for finding the appropriate technique for preparing electrodes with the highest specific capacitance, the presented studies showed that MOF-5 can be produced from the converted toxic waste. For example, by using DMF (from the synthesis of MOF-5, after recycling it by distillation) and terephthalic acid recovered from PET bottles. CMOF-5 could be also successfully reused from previously used electrodes after the intense acetone purification from the binder residues. In summary, CMOF-5 not only has a high potential to be used for super capacitance but is also easily manufactured from pristine and recycled substrates, which is what makes this material promising in industrial applications.

## Figures and Tables

**Table 1:** The comparison of the capacitance properties between the literature and our carbonized MOF-5.

Sample	Additives	Electrolyte	Electrode System	Composite Capacitance	Current Density/Scan rate	Reference
NPC650	-	1 M H_2_SO_4_	two	222 F/g	50 mA/g	[8]
PVDF-bonded carbon PVA-bonded carbon	binder: 10% of PVDF binder: 10% of PVA	0.5 M KCl	three	74.4–80.3 F/g 89.6–99.8 F/g	5 mV/s	[37]
Porous carbon	binder: 5% of PTFE conductive agent: 10% of acetylene black	1 M NEt_4_BF_4_	two	175 F/g	0.6 A/g	[41]
CZIF69a	-	0.5 M H_2_SO_4_	three	168 F/g	5 mV/s	[42]
CIRMOF-3-950	binder: 5% of PVDF conductive agent: 5% of carbon black	1 M H_2_SO_4_	two	239 F/g	5 mV/s	[43]
NPC800	binder: 10% of PTFE conductive agent: 10% of acetylene black	6 M KOH	three	226.6 F/g	1 A/g	[44]
UCN-20-750	binder: 10% of PTFE conductive agent: 10% of graphite	6 M KOH	three	256 F/g	1 A/g	[45]
CMOF-5-80	binder: 20% of PVDF	1 M H_2_SO_4_	two	218 F/g	1 A/g	this work

## Supplementary Materials

The following are available online at http://www.mdpi.com/2079-4991/8/11/890/s1, Figure S1: Higher scanning electron microscope (SEM) images magnification of the electrodes surface: A—carbonized Zn_4_O(1,4-benzodicarboxylic acid) (MOF-5) and poly(vinylidene fluoride) (PVDF) compressed pellet; B—PVDF membrane prepared by evaporation; C—PVDF membrane and carbonized MOF-5 prepared by evaporation, Figure S2: Isotherms of the electrodes from the carbonized MOF-5 and PVDF prepared by evaporation (A) and pellet compressing (B), Figure S3: Energy-dispersive X-ray spectroscopy (EDS) mapping of the copper (purple) and potassium (green) elements in the electrodes cross-section measured away and at potassium hydroxide wetting point, Figure S4: Nyquist plots (full and selected range) of the supercapacitros prepared with different binder:active material (MOF-5:PVDF) ratio, Figure S5: SEM images of MOF-5 synthesised from the distilled N,N-dimethylformamide (DMF) (A) and recycled terephthalic acid (B).
