# Supplementary material for: Poly(vinylidene fluoride) and Carbon Derivative Structures from Eco-Friendly MOF-5 for Supercapacitor Electrode Preparation with Improved Electrochemical Performance
Source: Nanomaterials (Basel). 2018 Nov 1;8(11):890. doi: 10.3390/nano8110890 (PMC6265847; doi:10.3390/nano8110890)
Supplement: Supplementary file 1 [file nanomaterials-08-00890-s001.pdf]

Supporting Information

Poly(vinylidene fluoride) and Carbon Derivative Structures from Eco-friendly MOF-5 for Supercapacitor Electrode Preparation with Improved Electrochemical Performance

Krzysztof Cendrowski *, Wojciech Kukulka, Tomasz Kedzierski, Shuai Zhang and Ewa Mijowska*

Nanomaterials Physicochemistry Department, Faculty of Chemical Technology and Engineering, West Pomeranian University of Technology, Szczecin, Piastów Ave. 42, 71-065 Szczecin, Poland; wojciech_kukulka@zut.edu.pl (W.K.); tomek_k95@o2.pl (T.K.); Shuai.Zhang@zut.edu.pl (S.Z.)

* Correspondence: kcendrowski@zut.edu.pl (K.C.); emjowska@zut.edu.pl (E.M.)

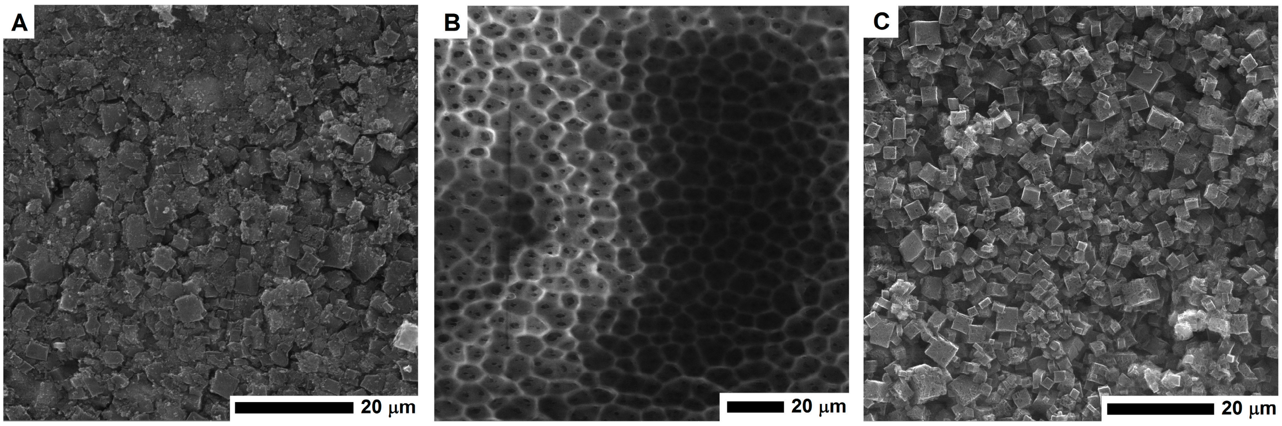

Figure S1. Higher scanning electron microscope (SEM) images magnification of the electrodes surface: **A**—carbonized Zn_4O(1,4-benzodicarboxylic acid) (MOF-5) and poly(vinylidene fluoride) (PVDF) compressed pellet; **B**—PVDF membrane prepared by evaporation; **C**—PVDF membrane and carbonized MOF-5 prepared by evaporation.

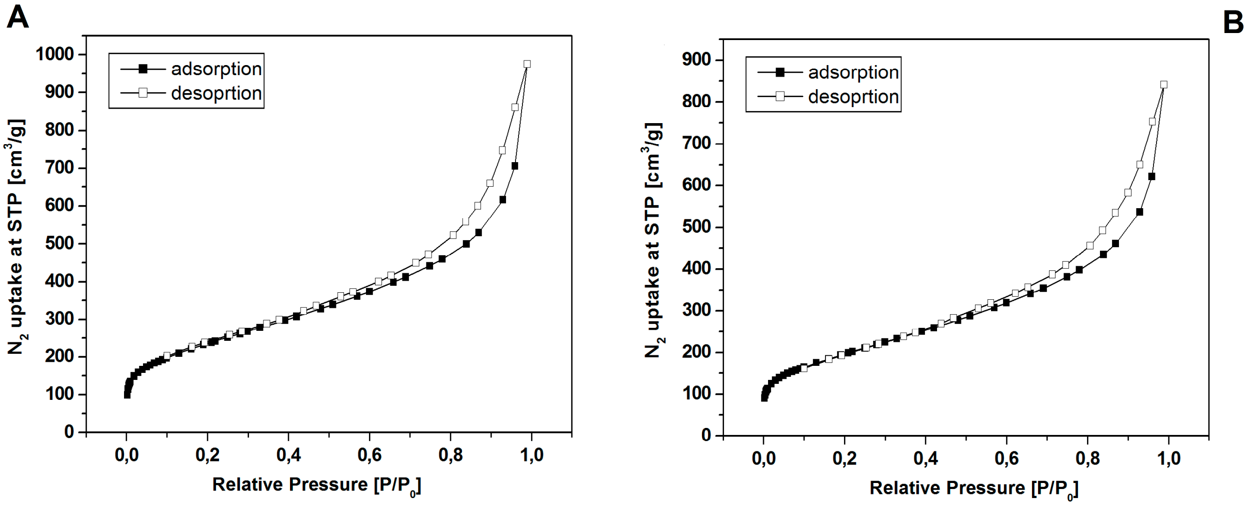

Figure S2. Isotherms of the electrodes from the carbonized MOF-5 and PVDF prepared by evaporation (**A**) and pellet compressing (**B**).

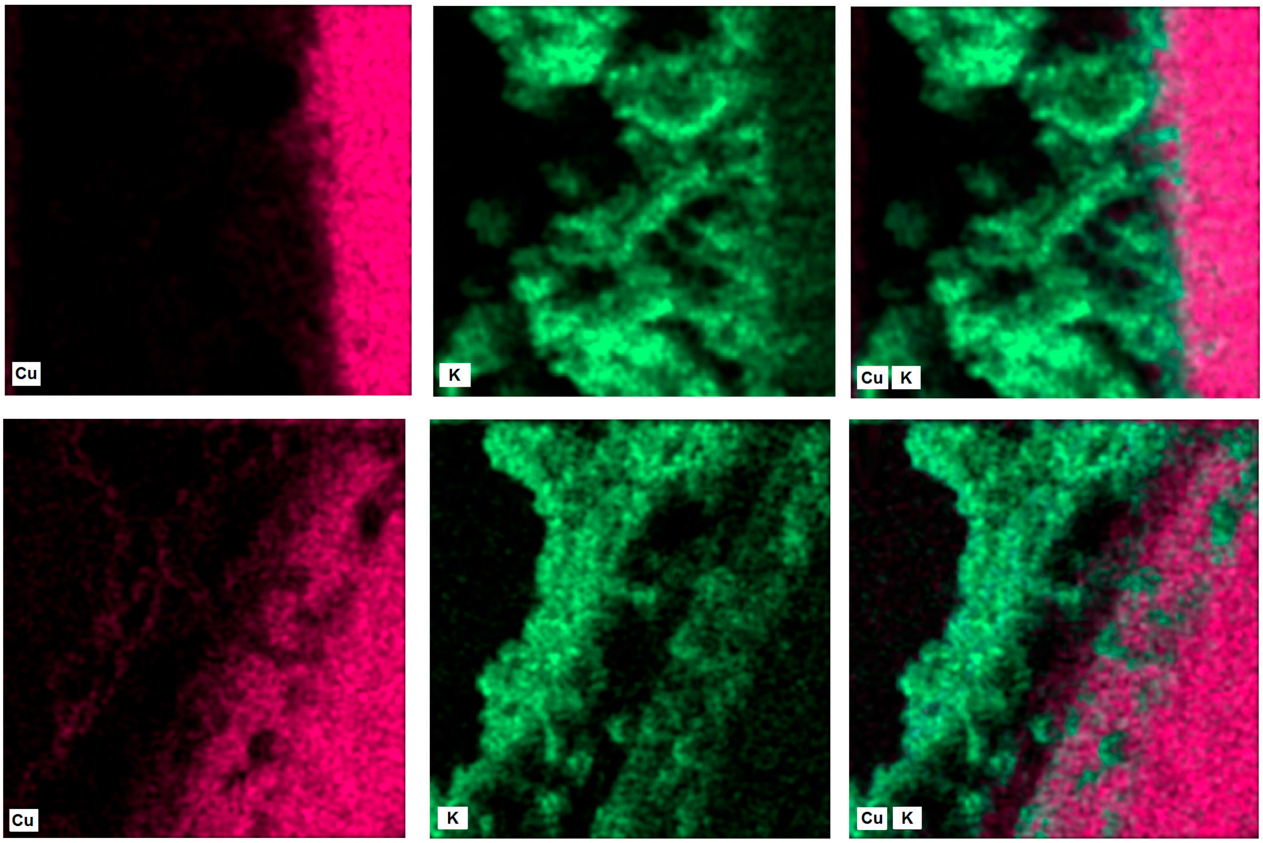

Figure S3. Energy-dispersive X-ray spectroscopy (EDS) mapping of the copper (purple) and potassium (green) elements in the electrodes cross-section measured away and at potassium hydroxide wetting point.

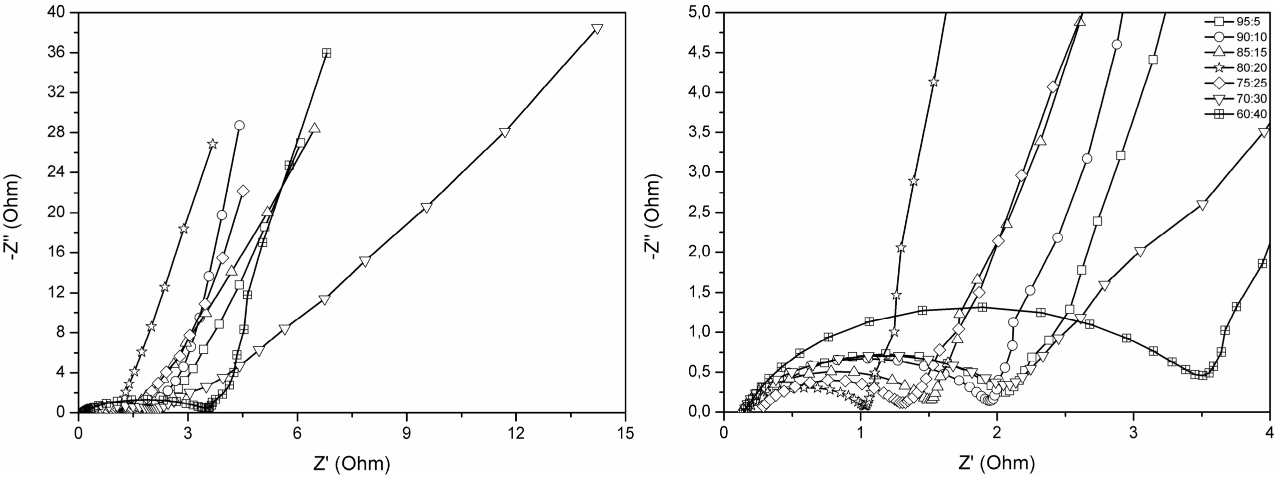

Figure S4. Nyquist plots (full and selected range) of the supercapacitros prepared with different binder:active material (MOF-5:PVDF) ratio.

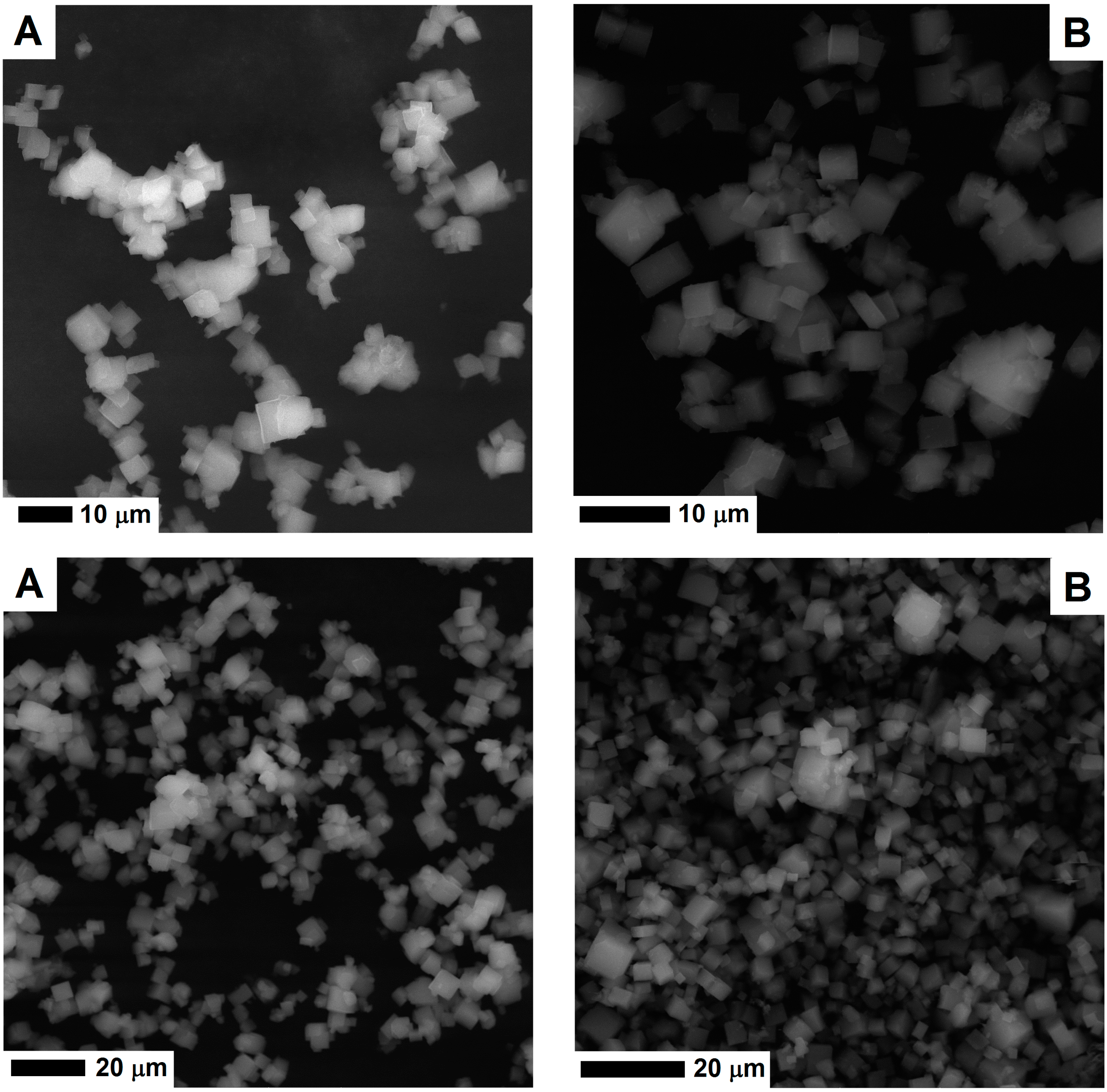

Figure S5. SEM images of MOF-5 synthesised from the distilled N,N-dimethylformamide (DMF) (**A**) and recycled terephthalic acid (**B**).
